# Determining the Level of Threat in Maritime Navigation Based on the Detection of Small Floating Objects with Deep Neural Networks

**DOI:** 10.3390/s24237505

**Published:** 2024-11-25

**Authors:** Mirosław Łącki

**Affiliations:** Faculty of Navigation, Gdynia Maritime University, 81-225 Gdynia, Poland; m.lacki@wn.umg.edu.pl

**Keywords:** deep neural networks, detection and classification, safety of marine navigation, image processing, object detection techniques

## Abstract

The article describes the use of deep neural networks to detect small floating objects located in a vessel’s path. The research aimed to evaluate the performance of deep neural networks by classifying sea surface images and assigning the level of threat resulting from the detection of objects floating on the water, such as fishing nets, plastic debris, or buoys. Such a solution could function as a decision support system capable of detecting and informing the watch officer or helmsman about possible threats and reducing the risk of overlooking them at a critical moment. Several neural network structures were compared to find the most efficient solution, taking into account the speed and efficiency of network training and its performance during testing. Additional time measurements have been made to test the real-time capabilities of the system. The research results confirm that it is possible to create a practical lightweight detection system with convolutional neural networks that calculates safety level in real time.

## 1. Introduction

The safety of marine navigation directly impacts the protection of human lives, the environment, and valuable cargo. Key factors in maintaining navigational safety include accurate positioning, effective communication, understanding of environmental conditions, and navigational situation.

Threats to marine navigation are numerous and diverse. They range from natural hazards like severe weather, rough seas, and poor visibility to man-made dangers such as collisions with other vessels, grounding on reefs or sandbars, and accidents in busy ports or narrow channels. In addition to these traditional threats, the increasing presence of floating objects poses a significant risk. These hazards, often referred to as marine debris, include a wide range of items such as derelict fishing gear, rubber, textiles, metal, and various types of plastic waste. The accumulation of such debris in the oceans can lead to dangerous situations for vessels, especially in busy shipping lanes, coastal areas, and regions prone to natural disasters.

Plastic debris and other floating objects can cause severe damage to a vessel’s hull, propellers, and rudders if collided with, potentially leading to accidents, grounding, or even sinking. These hazards can also obstruct critical navigation channels, disrupt traffic, and create challenges for search and rescue operations. Furthermore, smaller vessels and recreational boats are particularly vulnerable to these risks, as they may lack the robust detection and avoidance systems found on larger ships, that can integrate satellite imagery, oceanographic models, and reports from other vessels and from citizen science programs [1]. Thus, the safety of smaller vessels depends mostly on continuous visual observation.

Constantly watching for floating obstacles in the course of a vessel is a challenging and complex task. Several factors make this task difficult:

1. Visibility limitations: Floating objects, especially smaller ones like plastic debris or partially submerged containers, can be difficult to spot, particularly in poor-visibility conditions such as fog, rain, or darkness. Even in daylight, the vastness of the open ocean can make it nearly impossible to detect all potential hazards in a vessel’s path.
2. Human limitations: Maintaining continuous vigilance for floating obstacles requires intense concentration and can be mentally and physically exhausting for the crew. Human error, fatigue, and distraction are significant factors that can reduce the effectiveness of manual monitoring.
3. Unpredictability of debris: The location and movement of floating debris are often unpredictable. Currents, tides, and winds can disperse debris across large areas, making it difficult to anticipate where these objects might be encountered. Additionally, debris can move rapidly, especially in rough seas, increasing the challenge of detecting and avoiding it.
4. Technology gaps: While radar and sonar systems can detect larger objects, they are less effective at identifying smaller or partially submerged debris. Some modern vessels are equipped with advanced optical sensors or infrared cameras, but these technologies also have limitations, particularly in adverse weather or lighting conditions, and require constant attention if not integrated with an automated detection system.

**Figure 1:**
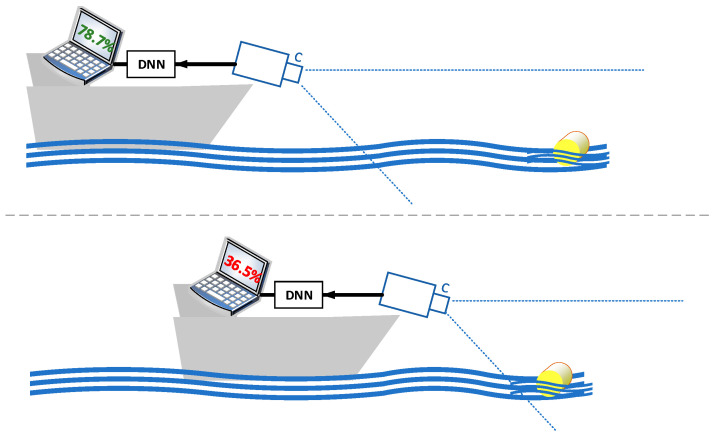
General proposal of a system calculating the level of safety regarding the distance from detected floating objects with usage of deep neural network.

Given these challenges, relying solely on human watchkeepers or traditional navigation tools is not sufficient to ensure safety. This is where a system based on deep neural network architecture (DNN) may be useful. The proposed solution can provide automated alerts, as shown on Figure 1, and enhance a crew’s ability to detect and avoid floating obstacles.

There are two main DNN structures considered in this study: Fully Connected (Dense) Neural Network (NN) and Convolutional Neural Network (CNN). In Dense NN each neuron in one layer is connected to every neuron in the subsequent layer. Dense net-works typically require a large amount of data and computational resources to train effectively and are sensitive to overfitting. Standalone large Dense NN is not as efficient as smaller Dense NN combined into convolutional architecture [2].

Convolutional Neural Networks (CNNs) are a type of deep learning algorithm that have been widely used in image object recognition tasks [3,4,5]. CNNs are specifically designed to deal with the variability of two-dimensional shapes and have shown superior performance compared to other techniques. CNNs are a powerful tool for image object recognition and classification. They are capable of extracting meaningful features from images and have been successfully applied in various domains, including medical imaging, activity recognition, real-time remote sensing monitoring, and big data analysis.

According to reviews of deep learning (DL) methods, there are several hundred important articles describing concepts and applications [6]. An example of the successful application of CNNs in image object recognition is the Inception architecture proposed by Szegedy et al. [7]. This architecture achieved state-of-the-art performance in the ImageNet Large-Scale Visual Recognition Challenge 2014 (ILSVRC14), which is a benchmark dataset for image classification and detection tasks. Inception architecture utilizes multiple parallel convolutional layers with different filter sizes to capture features at different scales, allowing for more accurate recognition of objects in images. Another example is the use of CNNs in medical image recognition. Here CNNs were applied to train medical images, such as magnetic resonance imaging [8], invasive ductal carcinoma [9], and computed tomography images [10], and achieved higher recognition rates compared to traditional methods. Furthermore, CNNs have been applied in various fields beyond image recognition. For example, CNNs have been used in recognizing human activity using wearable sensors [11] or the seismic facies classification [12].

Another interesting approach to image classification solution is deep transfer learning (DTL). This is a machine learning approach that enables models trained on one task to be adapted and applied to a related but different task. It leverages the pre-trained knowledge from a source domain (such as images, text, or other data) to improve performance on a new target domain, especially when labeled data for the target domain are limited or costly to obtain. This approach overcomes the drawbacks of traditional machine learning, in which the training datasets are separated and used individually for each task. A complete survey with detailed classification of DTL models has been presented in [13].

The novelty of this study is in its ability to calculate threat level for a vessel regarding the influence of detected floating objects on the safety of navigation. The practical application may be created in the future as a navigational support system for watch officers or helmsmen, generating warnings easy to read as threat levels calculated by trained neural networks. Other studies in the field of small floating object detection focus mainly on proper and fast identification and classification [14], also in low-light conditions [15]; thus the goals of the other studies are slightly different. The contribution of this study is in the design and comparison of a few different network topologies and selection of the best ones that fit the ‘lightweight’ category.

The structure of the paper is as follows: After this introduction, there is Section 2 de-scribing materials and methods used in this study. The next chapter provides simulation results for 12 neural networks of different topologies. The results are discussed in Section 4, and Section 5 consists of a brief summary and conclusions.

## 2. Materials and Methods

**Figure 2:**
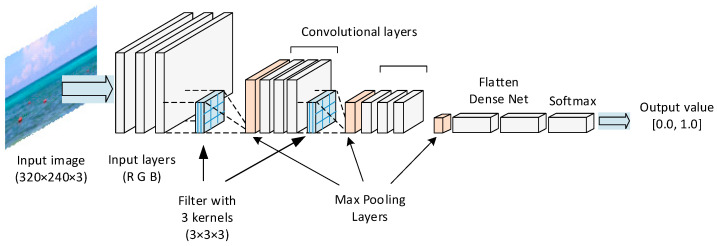
General architecture of CNN used in this study.

The architecture of a CNN consists of multiple layers, including convolutional layers, pooling layers, and fully connected dense layers (Figure 2). Input image is divided into three separate color channel input layers.

Next, the filters are applied to the input layers to extract local features. These filters, each 3 × 3 in size, are learned through the training process and can capture different patterns and textures in the image [7]. The pooling layers, introduced in 1990 by Yamaguchi et al. [16], downsample the feature maps to reduce the spatial dimensions and extract the most important features. The size of the pooling layers in this study is 2 × 2. The result of each convolutional layer is calculated by ReLu (rectified linear unit) activation function, which replaces each negative value by zero according to Equation (1):

f(x) = max(0, x)(1)

where x is the value from neurons of convolutional layer. The fully connected dense layers combine the extracted features and make safety calculations based on them [17]. The last dense network output is calculated with softmax function

(2) $$s\left(x_{i}\right)=\frac{e^{x_{i}}}{\sum_{j=1}^{n}e^{x_{j}}}$$

which transforms a vector of real numbers into a vector of probabilities.

**Figure 3:**
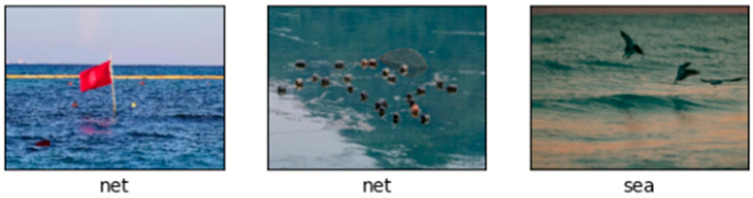
Examples of input images.

The images, required to train neural networks and validate them, were acquired from the following free image and video depositories: depositphotos.com, pexels.com, istockphoto.com, and stockvault.net. The examples of the images are shown on Figure 3.

The training set contains 200 images divided into two categories:

1. Clear water surface (tag: sea)—this collection contains photos of a calm and a stormy sea, with different daylight conditions, sometimes with flying or floating birds;
2. Polluted water surface (tag: net)—this collection contains photos of different plastic debris, fishing gear, buoys, and similar small floating objects.

The validation set was divided into the same categories as the training set. It consists of 280 images, completely different from the training set to observe possible overfitting. Overfitting occurs when a model learns to perform very well on the training data but fails to generalize to new, unseen data. Overfitting often occurs when the model is too complex relative to the amount of training data. In CNNs this could mean having too many layers, filters, or parameters relative to the size and diversity of the training dataset. An overfitted model fails to make correct predictions on data that it hasn’t encountered before. To prevent overfitting, the dropout layer has been added after the dense layer. Dropout randomly turns off neurons during training with probability 0.5 for each neuron.

Additional training parameters and limitations:

- 30 epochs of training;
- Each training batch consists of 30 input images;
- Each image is resized to 320 × 240 px
- The result is divided into two crisp categories, depending on network output value: not safe [0–0.5) and safe [0.5–1];
- Average accepted performance for each NN not less than 80% during training and validation;
- Size of the network not more than 1 GB, for lightweight property requirements.

## 3. Results

This section provides a description of the experimental results of 12 different DNN architectures and their interpretation.

Table 1 shows the basic parameters of each network. The numbers for the CNN layers are the numbers of filters, and the numbers for the Dense layers are the number of neurons.

The basic criteria for efficient network are an accuracy of at least 0.8 in both training and validation. The first four networks are unable to achieve good validation results and perform very poorly during training. Dense architecture requires many more neurons to operate effectively, which causes its size to significantly exceed the allowable limit of 1 GB.

**Figure 4:**
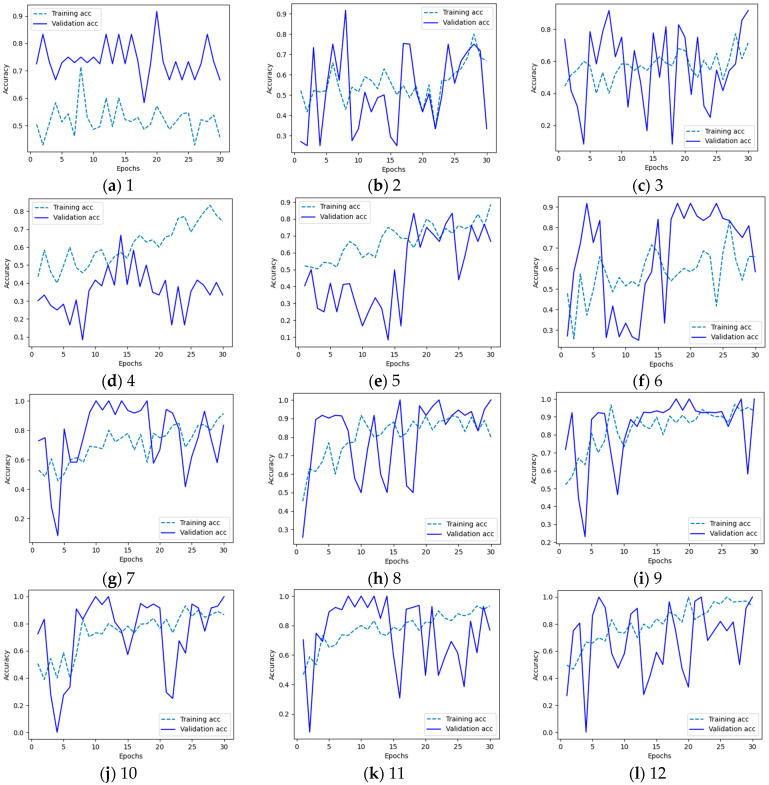
Training (dashed) and validation (solid) results for each network: (**a**) Network no 1 shows huge underfitting gap and is unable to learn effectively; (**b**–**f**) Networks 2–6 have big differences between training and validation values (**g**–**j**) Networks 7–10 perform well in comparison to other networks. (**k**,**l**) Networks 11 and 12 are also quite good, but due to overfitting occurrence and bigger size, they are not as good as smaller ones.

Although network 9 achieved better average results during training, network 10 has better results during validation and a smaller difference in values between the average result of training and validation (Figure 4). The size of network no. 10 is also less than 400 MB; therefore in this study, network no. 10 was selected as the best candidate for further tests and timing measurements.

Networks 11 and 12 show overfitting symptoms, and therefore are probably too big for this task. Additionally, network 12 is more than 1 GB in size, what makes it not as lightweight as expected.

Another important parameter that can help evaluate neural network performance is loss value. Loss is a measure of how well the network’s predictions match the actual target values. It quantifies the error between the predicted output and the true output during training. The goal of training a CNN is to minimize this loss value so that the network can make accurate predictions on new, unseen data. The loss function serves as a guide to adjust the weights and biases of the network. By calculating the loss after each forward pass, the network determines how far off its predictions are from the true values. Using this information, the network updates its parameters to reduce the error in future predictions.

In this research Binary Cross-Entropy (BCE), also known as Log Loss, has been used, with a formula as follows:

BCE = − (y × log(y_p_) + (1 − y) × log(1−y_p_)) (3)

where y is the true label, and y_p_ is the predicted label. This formula is applied for each training and validation step, and the total loss is usually averaged across all the examples in a batch. BCE is a commonly used loss function for binary classification tasks, where there are only two possible outcomes, as in this task the system classifies images as safe empty sea surface (labeled as “sea”) or detected floating objects (labelled as “net”). BCE measures the difference between the true label and the predicted probability from the network.

**Figure 5:**
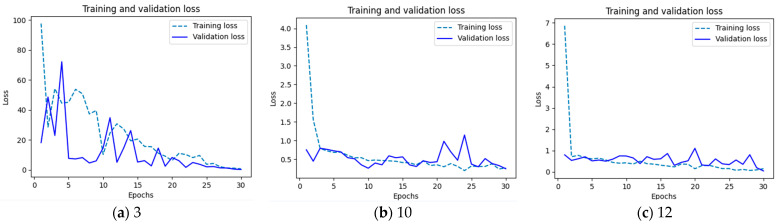
Examples of loss value for chosen networks: (**a**) Network no. 3 generates large loss values; (**b**) Network 10 has loss value about 0.5; (**c**) Network 12 has also values below 1, but there are some visible differences between training and validation values.

The examples of loss values are presented on Figure 5. 

During training, the loss value is often monitored over each epoch or batch. A decreasing loss value typically indicates that the model is learning and improving its performance. Overfitting can be detected if the training loss continues to decrease while the validation loss starts to increase. A low loss does not always correspond to high accuracy, and vice versa, as they provide different insights into the model’s performance.

**Figure 6:**
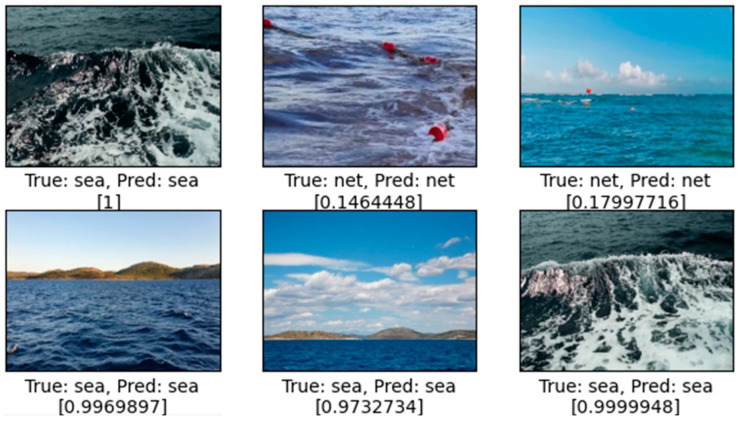
Examples of prediction results of network no. 10. Values in brackets closer to 1 indicate sea surface without floating objects.

Prediction examples shows that network no. 10 properly intentified clean sea surface and images with floating objects, as presented on Figure 6.

Additional time measurements have shown that the system is capable of processing about 20 images per second, which is sufficient and even excessive, because the expected target refresh of the threat level value in the final application will be no more than 2 times per second.

In this system, neural network calculations are performed entirely on the CPU. In the future, it could be considered to compare the performance of the hardware with hardware solutions using GPU and FPGA. At the current stage, however, the speed of the system using only the CPU is sufficient.

Downloading and saving images from the camera strongly depends on the hardware and software used. Cameras connected directly to a computer by cable can generate even several dozen images per second. The bottleneck of the transfer here may save individual images to the disk. In the tested system, the wireless camera connected via Wi-Fi allowed for recording 5 images per second at a resolution of 640 × 486 pixels (96 dpi). This was also a sufficient result for the smooth operation of the system.

## 4. Discussion

**Figure 7:**
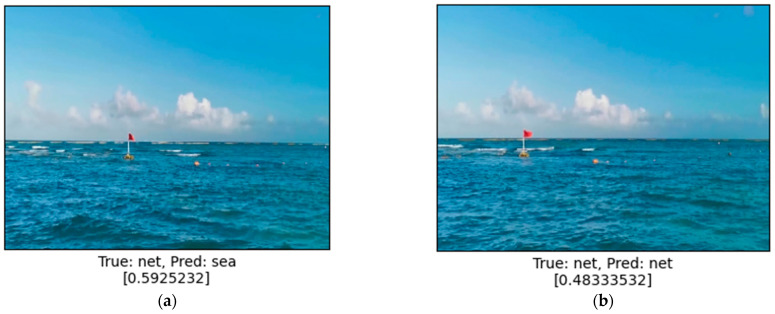
Examples of different prediction results of similar images: (**a**) A result above 0.5 indicates relatively safe situation; (**b**) A result below 0.5 is treated as not safe.

The obtained results of the system calculations are satisfactory, although in the case of estimating the level of safety or threat during navigation, using one strict threshold (e.g., value 0.5) is impractical. Classifying a given navigation situation only in two stages, as safe (predicted tag: sea) or dangerous (predicted tag: net), as shown on Figure 7, used for training neural networks is too restrictive. Therefore, in further studies, estimation using fuzzy values defining the ranges of the safety level will be considered.

Dividing the 0–1 range into several smaller intervals allows for a more accurate estimation of the threat level at a given moment, which will mainly depend on the distance from the detected object on the sea surface. In the current system, this can be realized at the system output in the end user interface, and from this point of view the system works properly.

**Figure 8:**
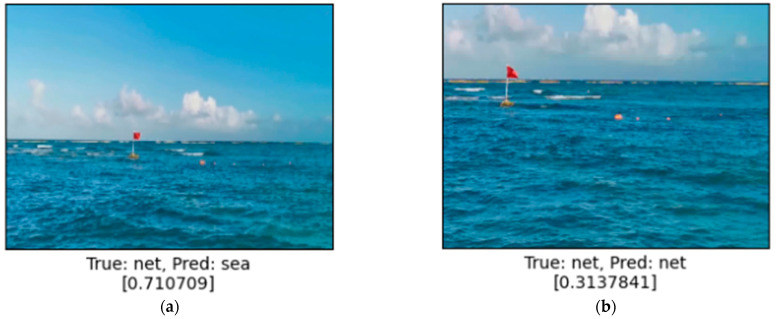
Comparison of network results depending on distance to floating objects. Range of values is a real value from 0 (not safe) to 1 (safe). Safety level in square brackets. Longer distance (**a**) qualifies situation as relatively safe (0.71). When vessel approaches a little closer (**b**) then safety level drops significantly to 0.31.

As can be seen in Figure 8, the longer the distance to the object, the higher the level of safety, and in Figure 8a the situation can be described as relatively safe, because the value calculated by the system is 0.71. The second situation in Figure 8b would require the watchman to take some action, because the detected objects are much closer and the system calculated the level of safety at about 31%.

Three subsets of safety levels may be distinguished:

- 0.0 to 0.4—not safe, taking action required;
- 0.4 to 0.6—relatively safe, but require intensive monitoring;
- 0.6 to 1.0—safe, some small object may be floating in long distance.

**Figure 9:**
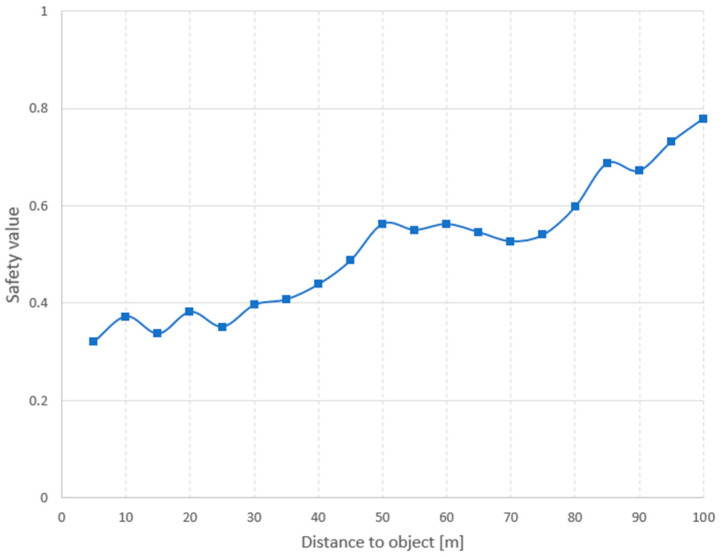
Safety level in relation to distance to floating object.

The safety level (Figure 9) and the number of subsets strictly depend on the distance to the detected floating objects, and to the type of vessels and their maneuvering characteristics. Additionally, the environmental conditions should be taken into account, i.e., visibility, wave height, wind, and other weather parameters.

Additionally, the system can be expanded to more precisely identify and mark objects on the image. One of the good solutions enabling real-time identification and classification of objects is the YOLO algorithm presented in 2015 [18]. The example of practical application of this algorithm for small-object detection in an underwater environment is described in [19]. The algorithm is still being developed and improved with new solutions that allow users to cope with some of the shortcomings of previous versions [20]. It is also worth noticing that each addition of new piece of visual or numerical information on the system screen may reduce readability or even overwhelm the user with information. Therefore, such auxiliary systems should be legible, and any additional elements should be optional and can be temporarily turned off.

## 5. Conclusions

The safety of marine navigation relies on a combination of skilled seamanship and technological advancements, including the effective use of decision support systems. The proposed solution is a good candidate that meets the conditions to become an easy-to-use lightweight application of the decision support system. Such a system during navigation through sea areas may help to assess safety level regarding the presence of small floating objects on the surface, difficult to spot in time without additional sensors other than the watchful eyes of the observer on board. Further practical research and measurements are needed to more thoroughly verify and test different navigation scenarios, especially in rough weather, low-light conditions, and high traffic.

## Figures and Tables

**Table 1:** Comparison of the size and average accuracy of the tested neural networks.

Network No.	CNN1	CNN2	CNN3	Dense	Size [MB]	Avg Test Accuracy	Avg Validation Accuracy
1	-	-	-	8	21.1	0.504	0.719
2	-	-	-	256	664.5	0.549	0.546
3	-	-	-	512	1310	0.598	0.598
4	2	-	-	8	3.4	0.664	0.483
5	2	4	-	16	3.34	0.648	0.727
6	2	4	8	32	3.16	0.63	0.734
7	4	8	16	64	12.5	0.816	0.778
8	8	16	32	128	49.9	0.86	0.851
9	16	32	64	256	199.8	0.881	0.818
10	16	32	64	512	399.3	0.861	0.849
11	32	64	128	512	799.1	0.859	0.759
12	64	128	256	512	1560	0.858	0.716

## Data Availability

Data available at http://kpisk.umg.edu.pl/lacki/research/2024/ accessed on 21 November 2024
